# Remote Loading: The Missing Piece for Achieving High Drug Payload and Rapid Release in Polymeric Microbubbles

**DOI:** 10.3390/pharmaceutics15112550

**Published:** 2023-10-28

**Authors:** Ghazal Rastegar, Mohammad Musa Salman, Shashank R. Sirsi

**Affiliations:** Department of Bioengineering, Erik Johnson School of Engineering, The University of Texas at Dallas, Richardson, TX 75080, USA; ghazal.rastegar@utdallas.edu (G.R.); musa.salman@utdallas.edu (M.M.S.)

**Keywords:** drug delivery, microbubbles, polymeric microbubbles, remote loading, drug loading, drug release, doxorubicin loading

## Abstract

The use of drug-loaded microbubbles for targeted drug delivery, particularly in cancer treatment, has been extensively studied in recent years. However, the loading capacity of microbubbles has been limited due to their surface area. Typically, drug molecules are loaded on or within the shell, or drug-loaded nanoparticles are coated on the surfaces of microbubbles. To address this significant limitation, we have introduced a novel approach. For the first time, we employed a transmembrane ammonium sulfate and pH gradient to load doxorubicin in a crystallized form in the core of polymeric microcapsules. Subsequently, we created remotely loaded microbubbles (RLMBs) through the sublimation of the liquid core of the microcapsules. Remotely loaded microcapsules exhibited an 18-fold increase in drug payload compared with physically loaded microcapsules. Furthermore, we investigated the drug release of RLMBs when exposed to an ultrasound field. After 120 s, an impressive 82.4 ± 5.5% of the loaded doxorubicin was released, demonstrating the remarkable capability of remotely loaded microbubbles for on-demand drug release. This study is the first to report such microbubbles that enable rapid drug release from the core. This innovative technique holds great promise in enhancing drug loading capacity and advancing targeted drug delivery.

## 1. Introduction

Ultrasound (US)-responsive materials, and microbubbles (MBs) in particular, have been safely used for diagnostic applications in the past few decades [1,2,3,4,5] and are approved by the FDA for clinical imaging purposes. The potential of using microbubbles for drug delivery has been widely acknowledged in recent years as well [6,7,8,9,10]. However, traditional approaches to loading and delivering drugs via MBs have suffered from various limitations. Existing methods have low drug payload due to limited surface area, as well as high burst release when triggered. As a result, there is an urgent need to develop innovative strategies for achieving higher drug payloads with microcapsules while maintaining the ability to be acoustically responsive for US-mediated delivery.

The most common manner by which MBs have contributed to drug delivery is sonopermeation, which is the formation of transient and/or permanent pores in the tumor vasculature by opening tight junctions. Studies have categorically proven that sonopermeation leads to higher drug concentrations in the tumor microenvironment [11,12,13], thus increasing the therapeutic index. The full range of bioeffects that sonopermeation has on the body over extended periods is still not entirely understood [14], and remains the most significant drawback of this technique. However, newer strategies are being developed that do not necessarily rely on sonopermeation, but rather unload drugs within the vascular space and rely on diffusive forces to accumulate drugs in tissue. These strategies rely on external stimuli (light, heat, and sound) in order to rapidly induce the unpacking of encapsulated molecules [15,16,17,18]. Subsequently, the drug molecules penetrate through the endothelium layer into the tissue via Fickian diffusion [19].

**Figure 1:**
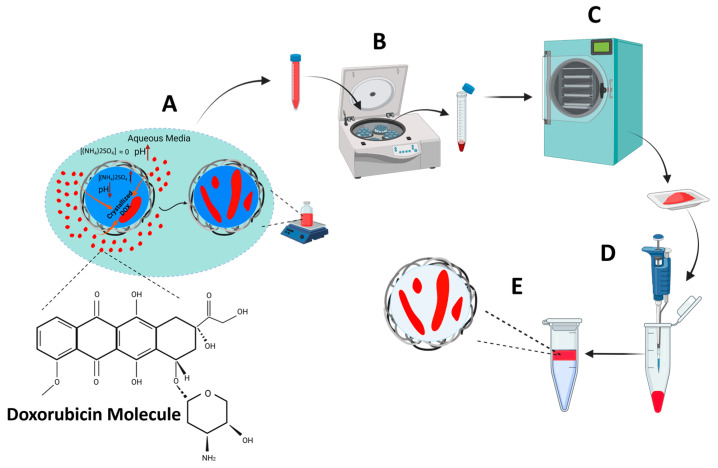
Experimental steps for producing RLMBs. (**A**) Loading DOX inside PLA MCs using an ammonium sulfate and pH gradient at 85 °C. Due to the presence of sulfate ions, doxorubicin precipitated and generates crystals of (DOX-NH_3_)_2_SO_4_. (**B**) RLMCs were separated from the unloaded drug by centrifugation (3200 RCF, 8 min). (**C**) RLMCs were lyophilized for 24 h, and the RLMB powder was collected. (**D**) Powder was resuspended in PBS. (**E**) RLMBs rose to the top of the tube. Created with Biorender.com (accessed on 15 July 2023).

Microbubbles are uniquely equipped to create effective unloading strategies. When exposed to ultrasound, the volumetric oscillation of MBs produces considerable shear stress and heat that can expedite their cargo release in circulation, resulting in a substantial local elevation of drug concentrations outside the carrier [20]. The key challenge has been to successfully add drugs to the MB construct so as to permit large payloads and rapid release at once. So far, there are two main strategies through which MBs have been used as drug carriers. First, drug molecules can be directly loaded on their surfaces or within their shells and then be released by an external ultrasound trigger. Second, MBs can be conjugated with drug-loaded liposomes, which have been frequently investigated for their drug release capabilities. Several studies regarding both loading approaches have shown positive results on drug release from MBs, although in both strategies, the MBs’ surface area limits the amount of drug payload they can carry. Thus, their overall drug payload falls short of expectations [17,21,22,23,24,25,26]. In order to improve the payload, we focused on a simple but effective remote loading strategy (incorporating a transmembrane gradient) to load the drug molecules within the cores of the vesicles, thus significantly increasing their drug-loading capacity since they are no longer limited by the vesicles’ surface areas. Enjoying a high loading capacity of DOX driven by a transmembrane ammonium sulfate gradient, Doxil^®^ was the first reported remotely loaded drug delivery carrier [24,25,26,27], and ever since, this loading mechanism has only been used to encapsulate small molecule drugs in liposomes [28,29,30,31,32]. Even though remote loading has been proven to be highly effective in liposomes, it has not been investigated on any other type of carrier. In this study, for the first time, we used a transmembrane ammonium sulfate and a pH gradient ([(NH_4_)_2_ SO_4_] _MC core_ >> [(NH_4_)_2_ SO_4_] _media_ and [H^+^]_MC Core_ >> [H^+^] _media_) to encapsulate DOX molecules in a crystallized form within the cores of polymeric microcapsules, and to subsequently turn them into polymeric MBs. A schematic of the experimental steps for producing remotely loaded microcapsules (RLMCs) and microbubbles (RLMBs) is presented in Figure 1. The RLMBs demonstrated high drug-loading capacity, low burst release, and rapid pressure-dependent DOX release in a US field. This is the first study to show rapid release of drug content from thick-shelled polymeric MBs.

## 2. Materials and Methods

### 2.1. Preparation of Polylactic Acid (PLA) Microcapsules (MCs)

MCs were created using a standard double emulsion technique [33]. Briefly, 0.5 g PLA (poly(L-lactic acid), viscosity~2.0 dL/g, 0.1% *w*/*v* in chloroform at 25 °C) was dissolved in 100 mL dichloromethane (both from Sigma-Aldrich, St. Louis, MO, USA), and then stirred for 1 h at 400 rpm. Ammonium sulfate (Sigma-Aldrich, St. Louis, MO, USA) solution (1 mL, 0.3 M, pH = 4.5) was added to the organic media, and the whole mixture was sonicated for 20 s using a probe tip Sonicator (Branson 450 Ultrasonics Sonifier with microtip attachment, Emerson, St. Louis, MO, USA) at 100% power. A second emulsion was formed by adding the first emulsion to 50 mL of polyvinyl alcohol (Sigma-Aldrich, St. Louis, MO, USA). The final mixture was added to 100 mL isopropanol and stirred overnight in a fume hood. The MCs were separated from the solution by centrifugation at 3200 RCF for 8 min.

### 2.2. Drug Loading and Encapsulation Efficiency (EE%) Measurement

DOX-loaded MCs were prepared in two different ways: remotely and physically. In order to prepare remotely loaded MCs (RLMCs), 2 mL of deionized water was added to 10^8^ empty MCs, and the pH of the solution was raised to a certain value. A certain volume (12.5, 25, 50, 100, 200 μL) of a DOX (MedChemExpress, Monmouth Junction, NJ, USA) solution (20 mg/mL) was added to the MCs and incubated for 12 h at certain temperatures and 400 RPM. The physically loaded MCs (PLMC) were prepared using a method described elsewhere [34]. Briefly, during the preparation of MCs, instead of the 1 mL ammonium sulfate solution mentioned before, 1 mL of a 5 mg/mL DOX solution was added to the PLA and dichloromethane solution. The rest of the PLMC preparation process was conducted in the same way as that of empty MCs.

The amount of loaded DOX in RLMCs and PLMCs was quantified after dissolving the vesicles in DMSO, then measuring the fluorescent intensity of DOX using a plate reader (Synergy H4 Biotek, Winooski, VT, USA) at 480 nm and 580 nm excitation and emission wavelengths, respectively. RLMCs and PLMCs were separated from the unloaded DOX by centrifugation at 3200 RCF for 8 min, and subsequently, dichloromethane was used to extract the loaded DOX from their cores and shells. The amount of loaded DOX was calculated using a standard curve of DOX fluorescent intensity against known DOX concentrations (n = 3). Empty MCs were used as negative controls to monitor the fluorescent intensity of the background. The encapsulation efficiency was calculated using Equation (1).

EE% = (Loaded DOX/Total DOX) × 100 (1)

### 2.3. Effect of pH and Temperature on EE%

The drug loading process was performed at neutral (7) and basic (10.5) pH values and at temperatures of 25, 45, 65, 85, and 95 °C. The EE% was measured in each case to achieve the optimized pH and temperature for a higher drug payload.

### 2.4. Size Distribution and Characterization of RLMCs

A BX50 Upright Microscope (ACH 60X/0.80 ∞/0.17 objective) and a Rolera Bolt CMOS QImaging Camera (Surrey, BC, Canada) were used to visualize and image RLMCs, respectively. To prepare RLMCs for imaging, they were diluted to a certain point (final concentration of 5 × 10^7^ number/mL), pipetted on a 25 × 75 × 1 mm, and covered by a glass coverslip (Fisher Scientific, Waltham, MA, USA). The size distribution of RLMCs was investigated using a Multisizer 4e Coulter Counter (n = 3). Transmission electron microscopy (Jeol 1400+), with a voltage of 120 kV, was performed on RLMCs and empty MCs (control sample). Finally, 2 μL of a diluted sample was applied to a formvar-coated copper grid (Electron Microscopy Sciences) and dried using a nitrogen flow.

### 2.5. Preparation and Characterization of Remotely Loaded and Physically Loaded Microbubbles (RLMBs and PLMBs)

A sample of 10^8^ RLMCs or PLMCs was placed in liquid nitrogen for 10 s and then lyophilized for 24 h. The final powder was resuspended in 2 mL of phosphate buffer serum (PBS), then transferred to a 3 mL syringe. An Olympus FV3000RS confocal microscope was utilized for fluorescence imaging of RLMBs/PLMBs (both remotely and physically loaded) and empty MBs. The excitation and emission wavelengths used for visualizing DOX were 480 and 590 nm, respectively, and the final images were processed using ImageJ software (2.3.1). Bright field microscopy, TEM, and Multisizer 4e Coulter Counter were also used (same process as that applied to RLMCs) for characterizing RLMBs.

### 2.6. Burst Release Measurement of DOX in Response to Input Energy

RLMBs and RLMCs were vortexed at 3200 RPM for 5 s and at various time points (1, 5, 30, and 60 min). After that, the released DOX was quantified using the method mentioned previously. Release% was calculated using Equation (2). As a negative control, at the same time points, DOX release from non-vortexed RLMBs and RLMCs was investigated.

Release% = (Released DOX/Total loaded DOX) × 100(2)

### 2.7. Drug Release from RLMBs and RLMCs after Ultrasound Application

RLMBs (or RLMCs) were transferred to a Pasteur pipette and exposed to ultrasound (US) using a Sonoporator (3 MHz frequency, 2.5 W/cm^2^ intensity, and 10% duty cycle). The peak-negative pressures produced by the Sonoporator were 0.6 MPa and 2 MPa (using a magnifying lens). US was applied for 10, 30, 60, 90, and 120 s, and subsequently, the released DOX was quantified using the process described previously. As a negative control, DOX release from RLMBs that were not exposed to US was measured as well. As a positive control, complete destruction of RLMBs (100% drug release) was performed using an Ultrasonic Bath Sonicator (Fisher Scientific, Waltham, MA, USA).

## 3. Results and Discussion

### 3.1. Drug Loading and EE% Measurement and Optimization

The first aim of this study was to increase and optimize the EE% and the amount of loaded DOX in RLMCs, respectively. For this purpose, we used a transmembrane ammonium sulfate and pH gradient method ([(NH_4_)_2_SO_4_]_MC core_ >> [(NH_4_)_2_SO_4_] _media_ and [H^+^]_MC Core_ >> [H^+^] _media_). This loading strategy, also known as remote loading (or active loading), was first reported for liposomes [24] and has only, to the best of our knowledge, been applied to liposomes and polymersomes ever since. Here, for the first time, we report remote loading of DOX inside PLA MCs. Remote loading of DOX in the cores of MCs is technically a base exchange of DOX molecules (outside of MC core) with the ammonium ions (inside of MC core) [32]. Being a weak amphipathic base, DOX has the tendency to migrate toward a medium with a lower pH, in this case inside the MC core, thus becoming protonated and reaching a lower chemical potential [35]. Because of the presence of sulfate ions inside the MC core, DOX molecules precipitate and form a solid crystal ([DOX^+^]_2_SO_4_) [36].

We used a 0.3 M solution of ammonium sulfate with a pH value of 4.5 as the aqueous phase inside the RLMC core. A schematic of the loading process is shown in Figure 1. The pH value of the outside media was set at either a neutral or a basic value, 7 or 10.5, respectively, and its effect on EE% was monitored. pH = 10.5 was about one unit higher than the pKa of DOX, meaning that, according to Equation (3), at this pH level, there were 10 times more unprotonated DOX molecules than there were protonated ones, which was the reason for choosing a basic pH.

(3) $$pKa\cdot =\cdot pH\cdot +log\cdot \left(\frac{\left[DOX\right]}{\left[DOX^{+}\right]}\right)$$

**Figure 2:**
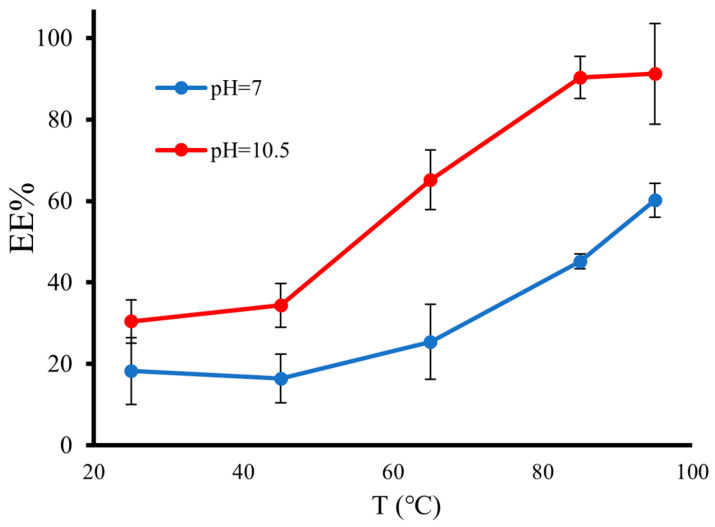
EE% of RLMCs at 25, 45, 65, 85, and 95 °C and at neutral and basic pH values (n = 3). T = 85 °C at pH = 10.5 was chosen as the optimum point, with EE > 90%. The EE% at T = 95 °C and pH = 10.5 was not significantly different from that at T = 85 °C and pH = 10.5 (*p* > 0.05, n = 3).

Another parameter that needed to be optimized for the purpose of reaching a higher EE% was the temperature of the experiment. For this, we carried out the 12-hour incubation process at 25, 45, 65, 85, and 95 °C, and at the pH values mentioned previously (7 and 10.5). The amount of initial DOX was 0.25 mg for 10^8^ empty MCs. All experiments were performed in triplicate. In Figure 2, the EE% against temperature at two different pH values is shown. As expected, in general, a higher pH resulted in a higher EE%. This is due to the number of unprotonated DOX molecules at pH = 10.5, which is significantly higher than that at pH = 7. In other words, at pH = 10.5, more DOX molecules had the tendency to pass through the MC shell towards a higher concentration of [H^+^], resulting in higher drug loading. Based on the results shown in Figure 2, temperature also has a direct correlation with EE%; at T ≥ PLA glass temperature (65 °C), the EE% significantly increased because the PLA shell became less rigid. At T = 85 and 95 °C (pH = 10.5), the EE% reached its highest value (>90%). However, there was no significant difference (*p* > 0.05) between their values; therefore, T = 85 °C was chosen as the optimum temperature because it led to less water evaporation during the 12-h experiment.

**Figure 3:**
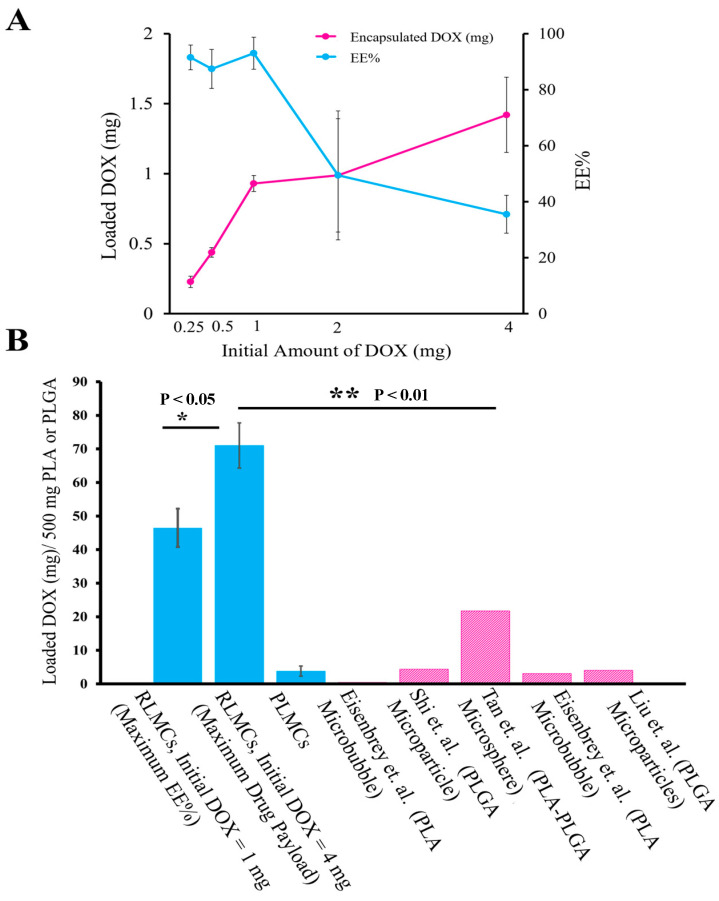
(**A**) EE% and amount of loaded DOX (mg) in 10^8^ RLMCs (n = 6). An initial DOX of 1 mg was chosen as the optimum drug concentration, whereas the maximum drug payload was achieved with 4 mg of initial DOX. (**B**) The amount of loaded drug (mg) per 500 mg of PLA/PLGA in previously reported carriers and that of RLMCs reported in this study [36,40,41,42,43].

The next step after optimizing the pH and temperature values was to optimize the amount of drug payload. For this purpose, we used various amounts of the initial drug, namely, 0.25, 0.5, 1, 2, and 4 mg DOX/10^8^ MCs. Based on the results shown in Figure 3A, at initial DOX = 1 mg, the highest EE%, and at initial DOX = 4 mg, the highest amount of loaded drug (mg), was achieved. At initial DOX = 4 mg, however, the EE% was significantly lower than that of initial DOX = 1 mg (less than 40% and higher than 90%, respectively), meaning that most of the initial drug would not be encapsulated, but rather wasted. Therefore, we chose initial DOX = 1 mg/10^8^ MCs as the optimum value of the initial drug and continued further experiments using this concentration. In Figure 3B, a comparison between the drug payload (mg) of RLMCs (those with the highest EE% and those with maximum drug payload) per 500 mg of PLA and that of previously reported DOX-loaded carriers per 500 mg PLA/PLGA is shown [33,37,38,39,40]. The Wheatly group reported DOX-loaded polylactic acid (PLA) MBs for “ultrasound-responsive drug delivery” [33]. The mechanism by which they generated the drug-loaded MBs was simple but effective [41], and they achieved 3.1 mg DOX/500 mg PLA drug payload and 75% drug release during 20 min of US exposure. For a better comparison, we also loaded DOX onto the surface of the MC shell using the same method. Herein, we refer to this method as “physical loading”. Based on the results presented in Figure 3B, the loading capacities of RLMCs with optimized EE% and maximum drug payload were more than 12-fold and 18-fold higher than that of physically loaded microcapsules (PLMCs), respectively. This demonstrates the significant role that remote loading plays when it comes to MCs’ drug payload.

### 3.2. Size Distribution and Characterization of RLMCs

**Figure 4:**
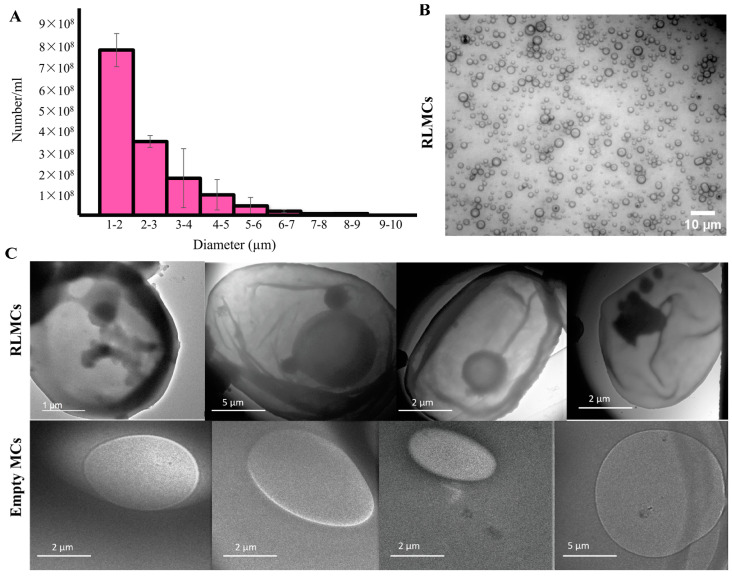
RLMC characterization (**A**) Size distribution of RLMCs (n = 3). The median diameter was 1.9 ± 0.8 μm. (**B**) Optical microscopy image of RLMCs. (**C**) TEM images of RLMCs (**top row**) and empty MCs (**bottom row**). Crystallized DOX can be observed within the cores of RLMCs using electron microscopy, but not optical microscopy, because of the lower wavelength of electrons (higher resolution) compared to that of light.

The size distribution of RLMCs was investigated using a Coulter counter and is represented in Figure 4A. The average concentration, median size, and mean diameter were 1.3 × 10^9^ ± 3.2 × 10^8^ MCs/mL, 1.9 ± 0.8 μm, and 2.2 ± 1.1 μm, respectively (n = 3). To verify the presence of crystallized DOX in the core, transmission electron microscopy (TEM) was performed on both RLMCs and empty MCs (negative control), the images of which are presented in Figure 4C. Non-crystallized DOX would not appear in the images, indicating that the black objects inside the core of RLMCs are, in fact, crystallized DOX. It is worth mentioning that the sizes of the MCs shown in the TEM images do not represent the actual size distribution of RLMCs, but rather a significantly higher size range. This is due to the fact that RLMCs with larger sizes were easier to image. The optical microscopy images (Figure 4B), on the other hand, are a proper representation of the actual size distribution of RLMCs.

### 3.3. Preparation and Characterization of RLMBs

**Figure 5:**
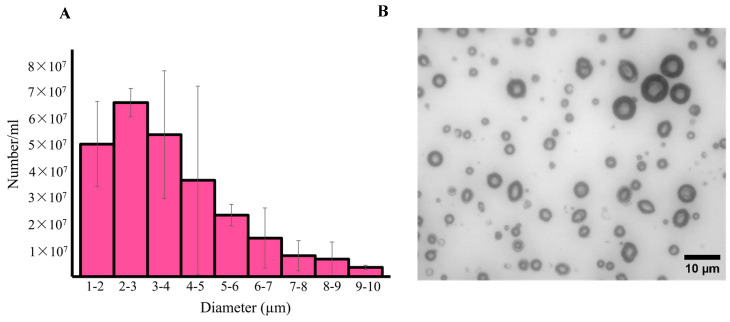

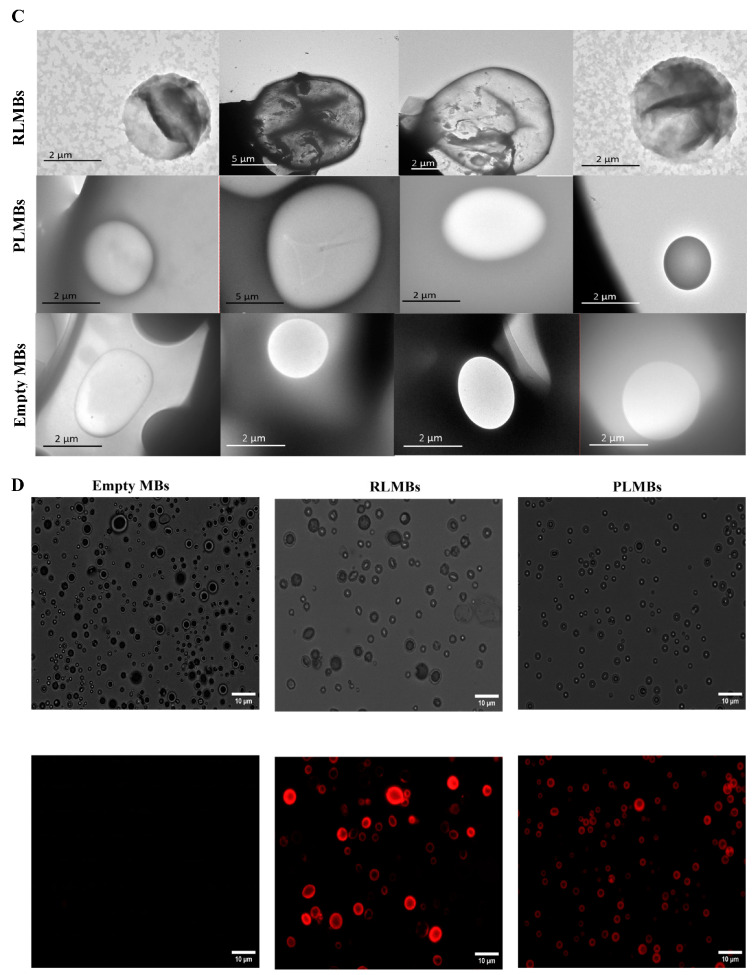
RLMB characterization. (**A**) Size distribution of RLMBs (n = 3). The median diameter was 3.2 ± 0.6 μm. (**B**) Optical microscopy image of RLMBs. (**C**) TEM images of RLMBs (**top row**), PLMBs (**middle row**), and empty MBs (**bottom row**). Crystallized DOX can be observed in remotely loaded MBs, but not in physically loaded MBs. (**D**) Confocal microscopy images of remotely loaded (**left**), physically loaded (**middle**), and empty MBs (**right**). In RLMBs, the crystallized DOX was highly quenched (more than 96% decrease in fluorescence intensity); hence, no visible fluorescence was detected from the core.

RLMCs/PLMCs were freeze-dried for 24 h; as a result, their liquid cores were sublimed and replaced with air. The solid crystallized DOX, on the other hand, remained intact. When resuspended in PBS, RLMBs/PLMBs were formed. The size distribution of RLMBs, similarly to that of RLMCs, was measured using a Coulter counter. The average concentration, median size, and average diameter were 2.4 × 10^8^ ± 3 × 10^7^ MBs/mL, 3.2 ± 0.6 μm, and 3.6 ± 0.9 μm, respectively (Figure 5A). Unlike RLMCs, however, RLMBs did not exhibit a normal size distribution (there is no peak in the graph), indicating a high dispersity in their size, which was also confirmed by optical microscopy imaging (Figure 5B). To verify the fact that crystallized DOX was intact and remaining in the core, TEM was performed on RLMBs and empty MBs (negative control). PLMBs were also imaged to compare their cores to those of RLMBs and to confirm that only remotely loaded vesicles demonstrated crystallized DOX within their core. TEM images are presented in Figure 5C. Similarly to that of MCs, the TEM images of MBs are not representations of their size distributions. Unlike empty MBs and PLMBs, DOX was observed inside the cores of RLMBs. To the best of our knowledge, this is the first report of MBs with drugs loaded within their core.

Since DOX has fluorescent properties, confocal microscopy (excitation wavelength = 480 nm, emission wavelength = 580 nm) was also performed on RLMBs, PLMBs, and empty MBs. As expected, no fluorescence was observed in the case of empty MBs. PLMBs, on the other hand, had fluorescent shells, indicating and confirming the fact that DOX was loaded in their shells. Similarly to PLMBs, RLMBs had fluorescent shells, but not fluorescent cores. The existence of DOX in the shells of RLMBs was due to the strong electrostatic attraction between DOX and PLA molecules [42]. In other words, during the drug loading process, when DOX molecules migrated through the PLA shell toward the MC core, a fraction of the DOX remained in the shell. The absence of fluorescence in the RLMB core was due to the fact that crystallized DOX is highly quenched. To confirm this, we measured the fluorescent intensity of DOX when loaded in RLMBs and extracted from MBs (using dichloromethane). The result (data not presented here) suggested that loaded (crystallized) DOX had approximately 96% less fluorescent intensity, which is consistent with results reported elsewhere [44]. Considering this, the fact that crystallized DOX is observed in TEM images but not in confocal images is understandable and expected.

### 3.4. Burst Release of DOX from RLMBs and RLMCs in Response to Input Energy

**Figure 6:**
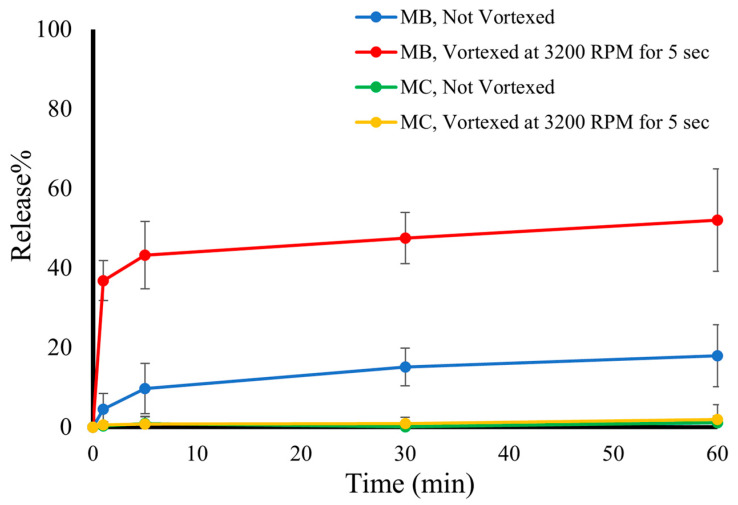
Drug Release% of RLMBs and RLMCs with and without input energy (being vortexed at 3200 RPM for 5 s). The data suggest that unlike MCs, MBs’ Release% significantly increased as a result of exposure to input energy.

After being resuspended in PBS, RLMBs were vortexed at 3200 RPM for 5 s, and the burst release of DOX at various time points within 60 min was measured. This was performed to evaluate the effect of input energy on the drug release of RLMBs. As a negative control, the burst release of RLMBs that were not vortexed was measured and compared with that of RLMBs. Based on the results shown in Figure 6, input energy had a noticeable effect on the burst release of RLMBs (more than twofold higher than that of non-vortexed RLMBs), which demonstrated a potential that Release% of RLMBs could be increased even more if higher amounts of energy (like US) were applied. The fact that RLMBs’ drug release might increase through this process convinced us to conduct the next set of experiments (Section 3.5). In the case of non-vortexed RLMBs, after 60 min, close to 20% of the total loaded DOX was released. We hypothesized that this was probably due to the burst release of the shell-loaded DOX. As mentioned previously, a fraction of the total loaded DOX resided within the PLA shell on the grounds of the electrostatic attraction between DOX and PLA molecules. The same process was performed on RLMCs as well. Figure. 6 suggests that both vortexed and non-vortexed RLMCs have negligible drug release after 60 min, which demonstrates that they are highly stable and not sensitive to input energy.

### 3.5. Drug Release from RLMBs and RLMCs after Applying Higher Amounts of Input Energy in the Form of Ultrasound

**Figure 7:**
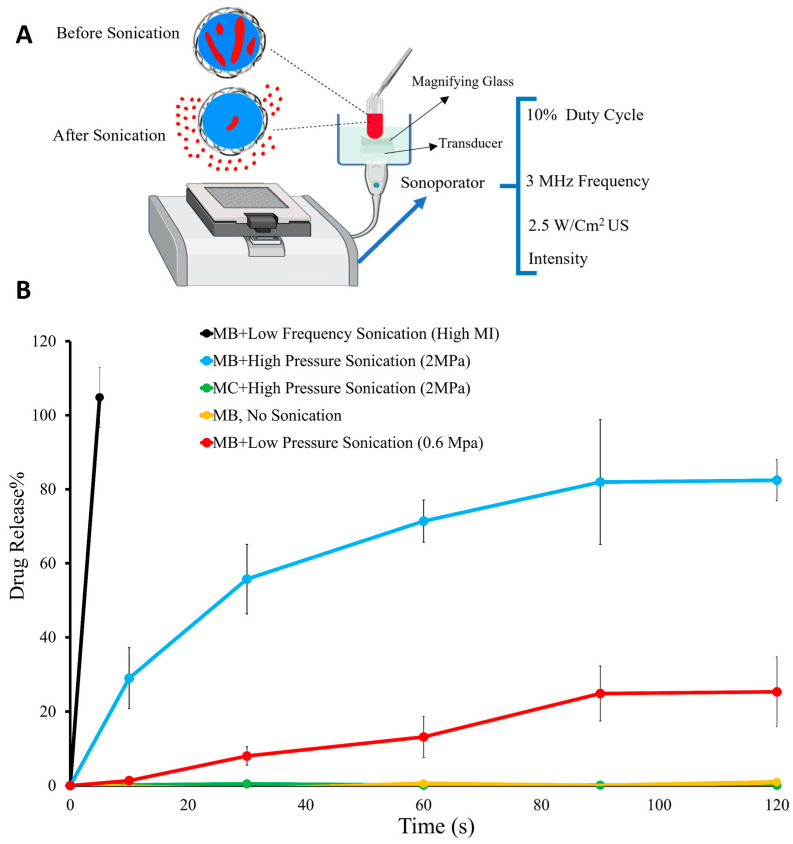
Drug release from RLMBs. (**A**) Schematic of the RLMB DOX release experimental setup. (**B**) Drug Release% of RLMBs after low frequency (black), low pressure (red), and high-pressure (blue) sonication and no sonication (green), as well as that of RLMCs after high-pressure sonication (yellow) (n = 3). Created with Biorender.com (accessed on 16 June 2023).

DOX release from RLMBs and RLMCs was investigated following US exposure for 10, 30, 60, 90, and 120 s. For this purpose, we used a sonoporator (3 MHz frequency, 2.5 W/cm^2^ US intensity, and 10% duty cycle) and a transducer. A schematic setup of this experiment is presented in Figure 7A. High and low peak-negative pressures were applied to release the drug. The default peak negative pressure of the instrument was 0.6 MPa (low pressure), but we were able to increase this value to 2 MPa using a magnifying glass on the transducer. Following US exposure, vesicles were separated from the solution by centrifugation, and the released DOX (in the solution) was quantified using a fluorescent plate reader (n = 3).

The Release% values of RLMBs and RLMCs are presented in Figure 7B. RLMCs exposed to high acoustic pressure demonstrated no drug release, which aligns with the results presented in the previous section (Figure 6). It seems that, in general, MCs are not a good option for drug release within a short period of time, since they are not sensitive to external energy exposure (due to their incompressible core). Even though RLMCs are capable of encapsulating a significant amount of the drug (more than 12-fold higher than previously reported similar DOX-loaded PLA carriers [40]), when it comes to “on demand” drug release, they do not lead to satisfactory results.

RLMBs, on the other hand, showed a pressure-dependent Release%. This is because their gas cores are US-responsive; when exposed to a US field, the gas core volume will oscillate. We hypothesized that this oscillation and the heat generated by it would facilitate the release of crystallized DOX. In general, without US exposure, no drug release from RLMBs was monitored. It is worth mentioning that the experiments reported in this section were conducted after those in Section 3.4. In other words, at t = 0, the burst release (no input energy) of RLMBs (Figure 6) had already occurred, and they were in a steady-state phase. For this section, Release% was calculated using Equation (4).

Release% = (released DOX/total remaining DOX after burst release) × 100 (4)

When exposed to low acoustic pressure (0.6 MPa), 25.3 ± 9.3% of DOX was released after 120 s, whereas 82.4 ± 5.5 Release% was achieved in response to a higher acoustic pressure (2 MPa). The DOX-loaded PLA MB reported by Eisenbrey et. al. (DOX was loaded on the surface of the MBs) released 75% of the cargo in 20 min (peak positive pressure of 1.68 MPa and peak negative pressure of 0.94) [33], indicating that RLMBs had drug release more than 10 times faster than the most similar previously reported carrier.

We also applied low-frequency US (40 kHz) to the RLMBs using a bath sonicator. Based on Equation (5), low frequency leads to high mechanical index (MI); we hypothesized that high MI would facilitate complete drug release from RLMBs in a short period of time. The result was quite promising, since after only 5 **s** of exposure, 100% of the DOX was released (Figure 7B).

(5) $$MI=\frac{P}{\sqrt{f}}$$

*P*: peak pressure (MPa) *f*: frequency (MHz)

Even though, to a high degree, we were able to improve drug release rates from RLMBs in vitro, additional testing using RLMBs in flow is warranted to mimic in vivo circulation. For this, RLMBs were exposed to US for 120 s continuously, which is difficult to achieve for circulating particles. Therefore, higher amplitudes of pressure or lower frequencies should be considered for in vivo drug delivery. Another possible approach that might lead to faster drug release (fast enough to be effective in vivo) is to manipulate the shell composition. For example, incorporating polymers with lower glass transition temperatures in the shell might lead to faster breakdown of the RLMBs in a US field, and, thus, faster drug release. Furthermore, reducing the thickness of the MBs could lead to faster cracking of the MB when exposed to US [43]. The hydrophobicity and molecular weight of the polymer have a significant role in the speed of drug release as well. Polymers with lower molecular weight and hydrophobicity (such as PLGA) have a lower glass transition temperature and less resistance against hydrolysis (MB degradation), respectively. For example, it has been reported that MBs with a 1:1 ratio of PLA to PLGA (low molecular weight) composition have significantly lower circulation time and could have faster drug release in a US field [45].

RLMBs have the potential to be highly effective in cancer therapy applications, even without immediate vascular uncaging. For instance, in chemoembolization therapy [46,47], RLMBs could accumulate in tumor arteries, providing a means by which to control drug release while also reducing the tumor’s blood supply. Other methods of directing the deposition of RLMBs could involve sonopermeation, which enhances the extravasation of circulating particles, or molecular targeting of RLMBs to tumor vasculature. Surface-loaded MBs have been utilized for this purpose thus far, delivering drugs and subsequently releasing them upon deposition to the vasculature through ultrasound exposure. This has yielded promising results [48,49,50].

## 4. Conclusions

In this study, we demonstrated a superior drug loading strategy for microbubble-targeted drug delivery. This strategy uses a transmembrane ammonium sulfate and pH gradient to load drugs in MCs. This is followed by subliming the liquid core, resulting in gas-filled microbubbles with crystallized doxorubicin in their cores. This method marks a significant departure from the conventional surface-loading or physical loading techniques. The loading capacities of RLMCs with optimized EE% and at maximum drug payload surpassed that of PLMCs by more than 12-fold and 18-fold, respectively. Importantly, the payload could be released into the solution upon ultrasound exposure, enabling better controlled drug release. RLMBs, therefore, have the potential to serve as next-generation ultrasound contrast agents for targeted and on-demand drug release applications.

## Data Availability

The data in this study are available on request from the corresponding author.

## Abbreviations

Abbreviation	Full name
US	Ultrasound
MB	Microbubble
DOX	Doxorubicin
RLMC	Remotely loaded microcapsule
RLMB	Remotely loaded microbubble
PLMC	Physically loaded microcapsules
PLMB	Physically loaded microbubbles
EE	Encapsulation efficiency
PLA	Polylactic acid
